# Task‐based detectability and angular‐sampling bounds on spatial resolution in cone‐beam micro‐CT: a closed‐form scan‐design analysis

**DOI:** 10.1002/mp.70737

**Published:** 2026-10-28

**Authors:** Falk L. Wiegmann, Nancy L. Ford

**Affiliations:** ^1^ Department of Physics and Astronomy The University of British Columbia Vancouver Canada; ^2^ Department of Oral Biological and Medical Sciences The University of British Columbia Vancouver Canada

**Keywords:** angular sampling, cone‐beam CT, Crowther criterion, ideal observer, micro‐CT, spatial resolution

## Abstract

**Background:**

Spatial resolution in preclinical cone‐beam micro‐CT is conventionally characterised by image‐domain metrics measured on a specific reconstruction of a specific phantom. Such metrics conflate resolution loss originating in the data (photon noise, angular undersampling) with resolution loss originating in the reconstruction (apodization, regularisation, voxel grid), and do not answer how much room is left for improvement on a given dataset.

**Purpose:**

To derive and evaluate two reconstruction‐independent resolution limits for preclinical cone‐beam micro‐CT, a photon‐noise limit from the projection‐domain signal‐known‐exactly, background‐known‐exactly (SKE/BKE) ideal observer with the Rose criterion, and an angular‐sampling limit from the Crowther criterion, and combine them into a single spatially varying picture that bounds what any classical reconstruction can achieve from a given scan.

**Methods:**

We derived closed‐form expressions for the photon‐noise minimum detectable disc diameter and an off‐axis fan‐beam extension, and combined them with the Crowther criterion via pointwise minimum. All resolution limits are theoretical evaluations of these closed forms for the geometry of a representative preclinical micro‐CT scanner. The only experimentally measured input is the unattenuated photon count per pixel, estimated from two flat‐field projections, and the exposure and projection‐count sweeps are hypothetical formula inputs, not acquired scans. The closed forms were validated by Poisson Monte Carlo simulation, and the specimen detectability map by fan‐beam forward projection of a previously acquired mouse attenuation map. The optimum split of a fixed total photon budget was derived analytically.

**Results:**

The combined limit yields a closed‐form scan‐design optimum. For the disc‐detection task, the maximum worst‐case spatial frequency scales as the cube root of the total photon budget per detector pixel (per‐view flux times number of views), and an analytic rule sets how to split that budget between flux and views. The off‐axis bound follows a “bowl” geometry rising from the centre to the rim, and the parametric sweeps show how each acquisition parameter reshapes this map. Simulated detectability agrees with the closed forms within 1% in minimum detectable diameter, on‐ and off‐axis, and forward‐projected real anatomy shifts the photon‐noise limit by only +3 to +8%, confirming the uniform specimen model is conservative.

**Conclusions:**

The two reconstruction‐independent limits combine into a usable engineering picture for scan design and detectability prediction in preclinical cone‐beam micro‐CT. The photon‐noise limit is a task‐based detectability threshold for a specified feature and contrast, expressed in frequency units rather than as a Fourier cutoff, and the combined map is a practical summary of these two independent, necessary constraints. Reconstructions that incorporate strong signal priors may exceed the Crowther limit at the cost of relying on those priors, but cannot exceed the photon‐noise limit at any cost. An accompanying open‐source interactive web calculator (https://ubc‐ford‐lab.github.io/ideal_observer_SKE_BKE_model/) implements these bounds for arbitrary scanner geometries.

## 1 INTRODUCTION

The spatial resolution of a CT scanner is usually characterised by image‐domain metrics such as the modulation transfer function (MTF), measured from a specific reconstruction of a specific phantom. These metrics are useful, but they conflate two different sources of resolution loss, ones that originate in the data (photon noise, angular undersampling) and ones that originate in the reconstruction (apodization, regularisation, voxel grid). Algorithm‐comparison studies in preclinical micro‐CBCT, including a recent characterisation of FDK filter kernels,^1^ report MTF values that vary by factors of two across reconstruction settings. None of them answer a more basic question. How much room is left to improve, given the data we already have?

The first bound on the answer is the projection‐domain ideal observer, which evaluates the matched‐filter detectability index $d^{\prime }$ of a known feature in known background.^2^, ^3^, ^4^ Under signal‐known‐exactly and background‐known‐exactly (SKE/BKE) assumptions, $d^{\prime }$ is a deterministic function of photon statistics and scan geometry,^5^, ^6^ with no dependence on the reconstruction algorithm. The Rose criterion ($d^{\prime }\geq 3$, or $d^{\prime }\geq 5$ in some recent work such as Hsieh et al.^4^) converts $d^{\prime }$ into a minimum detectable feature diameter $d_{\min}$,^7^ and via $f=1/(2d_{\min})$ into a spatial‐frequency limit. The bound is set by the input data. No reconstruction algorithm of any kind can exceed it.

The second bound is the Crowther criterion,^8^, ^9^, ^10^ which states that to reconstruct an image of radius r with spatial‐frequency content up to $f_{\max}$ requires at least $N_{\theta }=2\pi rf_{\max}$ angular samples. For a fixed number of projections $N_{\theta }$ acquired over an arc $\Delta \theta_{\text{span}}$, the spatial‐frequency limit at radial distance r from the rotation axis is $f_{\max}\left(r\right)=N_{\theta }/\left(2\Delta \theta_{\text{span}}\;r\right)$ (derived and reconciled with the preceding form in Section 2.2). This is a Nyquist bound on what an image‐domain observer can extract from the angular samples without additional prior information about the signal. Classical analytic reconstructions such as filtered backprojection (FBP) and the Feldkamp–Davis–Kress (FDK) cone‐beam algorithm, and iterative reconstructions without strong signal priors such as the simultaneous iterative reconstruction technique (SIRT) and ordered‐subset simultaneous algebraic reconstruction technique (OS‐SART), inherit it. Reconstructions that incorporate priors, such as neural networks trained on relevant data, implicit neural representations, and deep image priors,^11^, ^12^, ^13^, ^14^, ^15^ are not bound by Crowther and have been shown to recover frequencies above it. The cost is that they invoke assumptions about the signal that the Crowther bound does not. For the photon‐noise bound, the SKE/BKE matched filter is already the best possible decision rule for the detection task as posed, so prior‐based methods gain by injecting outside information or by changing the task, not by exceeding the data‐only statistical bound.

Both bounds sit inside the broader cascaded‐systems framework of medical‐physics image quality,^16^, ^17^, ^18^ which propagates signal and noise through every stage of the imaging chain to produce a frequency‐dependent noise‐equivalent quanta NEQ(f) and a task‐based detectability index $d^{\prime 2}=\int {\left|W\left(f\right)\right|}^{2}\;\mathrm{NEQ}\left(f\right)\;df$. The matched‐filter SKE/BKE bound used here is the linear shift‐invariant (LSI), stationary‐Gaussian, no‐anatomical‐clutter limit of that framework. Specifically, it is the projection‐domain limit of the cascaded NEQ in which detector blur, electronic noise, and reconstruction filtering have not yet entered the chain, leaving the input photon count $N_{0}$ as the only noise‐relevant input. The $d^{3}$ disc‐detection scaling we derive analytically below is the same scaling Gang et al.^19^ obtain via cascaded NEQ in cone‐beam CT. We chose the closed‐form simplification because it is evaluable from a handful of measurements rather than a full system MTF and noise‐power‐spectrum (NPS) characterisation, because it exposes scan‐design trade‐offs in analytic form, and because it keeps the angular‐sampling constraint (Crowther) separate from the photon‐noise constraint, which the integrated NEQ formulation tends to fold together.

This paper combines both limits into a single, spatially varying picture. We derive each bound for a circular feature in a uniform background, evaluate them on a GE eXplore CT 120 preclinical micro‐CT scanner, and present the combined‐limit map as the effective resolution ceiling for any classical reconstruction of these data. We then sweep the scan parameters that the experimenter controls, exposure and projection count, to show how each parameter reshapes the combined‐limit surface. Our specific aims are to:

1. Derive a closed‐form expression for the photon‐noise minimum detectable diameter $d_{\min}\left(\Delta \mu ,r\right)$ as a function of feature contrast and position, including an off‐axis fan‐beam extension that handles partial‐arc geometries, and validate both against Poisson Monte Carlo simulation of the projection‐domain ideal observer (Section 4.2).
2. Compose a combined‐limit map $f_{\text{eff}}\left(r\right)=\min\left[f_{\text{noise}}\left(r\right),f_{\text{Crowther}}\left(r\right)\right]$, with explicit treatment of its scope as a heuristic for the binding limit of classical reconstructions, and identify crossover contours that mark which limit dominates where (Section 4.5).
3. Use parametric sweeps over $N_{0}$ and $N_{\theta }$ to characterise how the combined‐limit surface deforms with each scan‐design choice, including the implications for trading dose against angular sampling (Sections 4.3 and 4.6).
4. Derive a closed‐form scan‐design optimum under a fixed total‐dose budget $\Phi =N_{0}N_{\theta }$, with the disc‐task dose‐resolution law $f_{\min}^{\max}\propto \Phi^{1/3}$ and the optimal‐allocation ridge $N_{\theta }^{★}\propto \sqrt{N_{0}}$ (Section 4.7).
5. Apply a region‐resolved detectability map to a real reconstructed mouse (*Mus musculus*) specimen (Section 5.2), with the per‐pixel transmitted counts recovered by forward‐projecting the anatomical attenuation map, showing how the bound can be sampled at every pixel of an FDK reconstruction to inform ROI selection and feature detectability for downstream analysis.

An open‐source interactive web calculator^20^ implements these bounds for arbitrary scanner parameters.

## 2 BACKGROUND

### 2.1 Projection‐domain ideal observer

Consider a circular disc of diameter d and linear‐attenuation contrast Δμ embedded in a uniform background of attenuation $\mu_{bg}$ and chord length L. The unattenuated photon count per detector pixel is $N_{0}$, the transmitted count through the background is $N_{bg}=N_{0}e^{-\mu_{bg}L}$, and for $N_{bg}\gg 1$ the photon noise variance per pixel in the log‐transformed projection is $\sigma_{p}^{2}=1/N_{bg}$. A ray at offset t from the disc centre passes through a chord of length $2\sqrt{R^{2}-t^{2}}$ (with R=d/2), so the disc contribution to the projection is

(1) $$\Delta p\left(t\right)=2\;\Delta \mu \sqrt{R^{2}-t^{2}},\;\left|t\right|<R,$$

and zero otherwise. Because the disc is circular, Δp(t) is identical at every projection angle.

The SKE/BKE ideal observer is the matched filter.^3^, ^4^ Its squared detectability index is the projection‐noise‐weighted sum of squared signal:

(2) $$d^{\prime 2}=\sum_{\theta_{j}}\sum_{i}\frac{{\left[\Delta p\left(t_{i}\right)\right]}^{2}}{\sigma_{p}^{2}},$$

where i indexes the detector‐pixel offset $t_{i}$ along the detector row and j indexes the projection angle $\theta_{j}$. Converting the pixel sum to an integral with detector pitch $\Delta a_{obj}$ at the object plane and using $\int_{-R}^{R}\left(R^{2}-t^{2}\right)\;dt=4R^{3}/3=d^{3}/6$:

(3) $$d^{\prime 2}=\frac{2\;N_{\theta }\;N_{bg}\;\Delta \mu^{2}\;d^{3}}{3\;\Delta a_{obj}}.$$

Setting $d^{\prime }=d_{\text{th}}^{\prime }$ (the detection threshold) gives the minimum detectable disc diameter

(4) $$d_{\min}={\left(\frac{3\;d_{\text{th}}^{\prime 2}\;\Delta a_{obj}}{2\;N_{\theta }\;N_{bg}\;\Delta \mu^{2}}\right)}^{1/3},$$

and the corresponding spatial‐frequency limit is

(5) $$f_{\text{noise}}=\frac{1}{2\;d_{\min}}.$$

Note that $f_{\text{noise}}$ is a task‐based detectability metric expressed in frequency units, not an image‐domain MTF cutoff. Through $d_{\min}$ it depends on the contrast and geometry of the specified feature, so a change in $f_{\text{noise}}$ reflects a change in the detectability of that feature, not a change in the spatial resolution of the imaging system. Section 5.1 discusses the formal status of this convention. Throughout we use the classical Rose threshold $d_{\text{th}}^{\prime }=3$.^21^ The stricter $d_{\text{th}}^{\prime }=5$ used in some recent work such as Hsieh et al.^4^ multiplies $d_{\min}$ by ${\left(25/9\right)}^{1/3}\approx 1.40$.

#### Off‐axis (fan‐beam) extension

For a feature at position r=(x,y) inside a phantom of radius $R_{ph}$ scanned by a point source at distance D from the isocentre, two quantities in Equation 3 become angle‐dependent. The chord through the phantom is

(6) $$\text{chord}\left(\theta ,r\right)=2\sqrt{{\left(S\left(\theta \right)\cdot \hat{d}\right)}^{2}-\left(D^{2}-R_{ph}^{2}\right)},$$

where S(θ)=(Dcosθ,Dsinθ) is the source position and $\hat{d}=\left(r-S\right)/\left|r-S\right|$. The local object‐plane pixel pitch also scales with source‐to‐feature distance,

(7) $$\Delta a_{obj}\left(r,\theta \right)=\Delta a_{obj}\cdot \frac{\left|r-S(\theta )\right|}{D}.$$

Folding both into Equation 3 gives

(8) $$d_{\min}\left(r\right)={\left(\frac{3\;d_{\text{th}}^{\prime 2}\;\Delta a_{obj}}{2\;\Delta \mu^{2}\;D\;\sum_{\theta_{j}}\frac{N_{0}\;e^{-\mu_{bg}\;\text{chord}\left(\theta_{j},r\right)}}{\left|r-S(\theta_{j})\right|}}\right)}^{1/3},$$

which reduces to Equation 4 at the isocentre and for full‐arc scans at large D. For partial‐arc acquisitions the map is no longer radially symmetric. The sum over $\theta_{j}$ runs over the $N_{\theta }$ acquired view angles and models the discrete acquisition sequence directly.

### 2.2 Angular sampling (Crowther criterion)

The Crowther criterion^8^, ^9^ bounds the spatial‐frequency content recoverable by a classical reconstruction (FBP, FDK, SIRT) from $N_{\theta }$ projections over an arc $\Delta \theta_{\text{span}}$. It follows from a Nyquist statement on the angular spacing of views. Successive views are separated by $\Delta \theta =\Delta \theta_{\text{span}}/N_{\theta }$, and between them a point at radial distance r from the rotation axis is displaced by an arc length rΔθ relative to the projection rays. Recovering a spatial frequency f without angular aliasing requires this displacement not to exceed half its period, rΔθ≤1/(2f). Solving for f gives the limit at radial distance r,

(9) $$f_{\text{Crowther}}\left(r\right)=\frac{N_{\theta }}{2\;\Delta \theta_{\text{span}}\;r}.$$

The bound diverges at the isocentre (r→0) but is capped by the detector Nyquist frequency $f_{\text{det}}=1/\left(2\Delta a_{obj}\right)$.

The familiar form $N_{\theta }=2\pi rf_{\max}$ quoted in Section 1 is Equation 9 with $\Delta \theta_{\text{span}}=\pi$, counting independent views over a nominal $180^{\circ }$ arc. The two forms are consistent for full‐circle acquisitions because opposing views measure conjugate rays of the same line integrals, so a 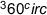 scan of $N_{\theta }$ total projections supplies only $N_{\theta }/2$ independent views over an effective span of π. Ray directions repeat modulo π, so Equation 9 is a criterion on the largest angular gap between sampled directions. For $\Delta \theta_{\text{span}}\geq \pi$ the conjugate‐covered band only revisits directions and never widens a gap, so the adjacent‐view spacing $\Delta \theta_{\text{span}}/N_{\theta }$ is exactly the binding quantity, including for the $192^{\circ }$ short scan used here. Below π the acquisition enters the classical limited‐angle regime where by the central‐slice theorem a directional wedge of width $\pi -\Delta \theta_{\text{span}}$ is never sampled, and frequencies whose directions fall inside it cannot be stably recovered without prior information.^22^ That wedge is then the largest gap between sampled directions, and applying the same half‐period condition to whichever gap is larger gives the conservative direction‐independent cap $f\leq 1/\;\left[2r\max(\Delta \theta_{\text{span}}/N_{\theta },\;\pi -\Delta \theta_{\text{span}})\right]$.

Equation 9 is a bound on what an image‐domain observer can extract from the projection‐angle samples alone, without prior information about the signal (Section 1 discusses reconstructions that incorporate priors and can therefore exceed it). The photon‐noise bound of Section 2.1 has no such restriction. It operates on the projection data before any reconstruction.

### 2.3 Combined effective limit

For a classical reconstruction, the effective spatial‐frequency limit at position r is the smaller of the two bounds:

(10) $$f_{\text{eff}}\left(r\right)=\min\;\left[f_{\text{noise}}\left(r\right),\;f_{\text{Crowther}}\left(r\right)\right].$$

Because $f_{\text{Crowther}}$ is a geometric Fourier‐sampling threshold while $f_{\text{noise}}$ is a task‐based detectability threshold, this pointwise minimum is a practical heuristic summary of two independent, necessary constraints rather than a unified frequency‐domain bound (Section 5.1). The locus where $f_{\text{noise}}=f_{\text{Crowther}}$ partitions the field of view into a noise‐dominated region (typically near the centre of attenuating phantoms, where $N_{bg}$ is small) and an angular‐dominated region (typically at the periphery, where r is large).

## 3 METHODS

### 3.1 Scanner and acquisition

The scanner modelled throughout is the GE eXplore CT 120 micro‐CT, operated at 80kV, 40mA, with 16ms exposure per frame, step‐and‐shoot rotation, and no frame averaging. The detector is a scintillator optically coupled to a CCD, providing 3500×2300 pixels at 0.0284mm effective pitch referred to the scintillator plane. A short‐scan acquisition ($192^{\circ }$, 220 projections) is assumed throughout. Table 1 lists the geometric parameters, which are taken from the scanner specification, not measured here.

**TABLE 1:** Scanner geometry parameters for the eXplore CT 120 used throughout.

Parameter	Value
Detector size (pixels)	3500×2300
Detector pixel pitch $\Delta a_{det}$ (mm)	0.0284
Source‐to‐detector distance SDD (mm)	451.5
Source‐to‐isocentre distance SOD (mm)	396.4
Magnification M= SDD/SOD	1.139
Object‐plane pixel pitch $\Delta a_{obj}$ (mm)	0.0249
Scan arc $\Delta \theta_{\text{span}}$	$192^{\circ }$
Number of projections $N_{\theta }$	220

We state the provenance of every dataset used here explicitly throughout. The only experimentally acquired data are two flat‐field projections used to estimate the unattenuated photon count $N_{0}$ (Section 3.2) and a slanted‐edge blade scan used to measure the presampling MTF reported in the Limitations (Section 5.3). Of these, only $N_{0}$ enters the model as an input. The in vivo mouse data underlying the detectability map of Section 5.2 are re‐used from previously published studies^23^, ^24^ and reconstructed with the Feldkamp–Davis–Kress (FDK) algorithm. No new object scans were acquired for this work. Everything else, every resolution limit, parametric sweep, and scan‐design optimum (Sections 4.1, [Link], Gap to actual reconstructions, Spatial structure of the limit, 4.2, 4.3, 4.4, 4.5, 4.6, 4.7), is a closed‐form theoretical evaluation of Equations (4), (5), (6), (7), (8), (9), (10) on a uniform water‐equivalent cylinder of radius R=13mm, a theoretical stand‐in for a typical mouse specimen that sets the chord length and background attenuation. Two numerical simulations validate the closed forms, a Poisson Monte Carlo simulation of the projection‐domain ideal observer (Section 3.5) and a fan‐beam forward projection of the re‐used mouse anatomy (Section 3.6).

### 3.2 Photon‐flux measurement

The unattenuated photon count per pixel $N_{0}$ was estimated from two flat‐field projections at the operating point of Table 1, with no object in the beam. After dark/bright correction, air pixels were pooled from across both frames. The variance of the half‐difference $\left(T_{1}-T_{2}\right)/\sqrt{2}$ removes correlated fixed‐pattern noise, leaving photon shot noise. Under Poisson statistics this gives

(11) $$N_{0}=\frac{1}{\text{Var}\;\left[\left(T_{1}-T_{2}\right)/\sqrt{2}\right]}.$$

We obtained $\text{Var}\left(T_{\text{air}}\right)=2.38\times 10^{-4}$, giving $N_{0}=4203$ photons/pixel, the spatial average across the detector. The statistical uncertainty of the estimator (spatial block bootstrap over the $1.6\times 10^{6}$ air pixels) is below 0.5%. The dominant uncertainty is the ±8% spatial variation of the incident beam (5th–95th percentile, beam profile and heel effect), which the single average absorbs rather than resolves. A pixel‐wise flat‐field calibration would yield $N_{0}\left(\theta_{j},r\right)$ but is not implemented here. Because $d_{\min}\propto N_{0}^{-1/3}$ (Equation 4), this spread corresponds to less than 3% in any reported $d_{\min}$.

### 3.3 Evaluation of the resolution‐limit surfaces

The on‐axis and Crowther bounds (Equations (4), (9)) are evaluated directly. The off‐axis map (Equation 8) is evaluated by summing $N_{bg}\left(\theta_{j},r\right)/\left|r-S\left(\theta_{j}\right)\right|$ over the 220 projection angles, on a 500×500 grid spanning ±47mm around the isocentre. The combined‐limit map (Equation 10) is the pointwise minimum of the two component maps. For the 3D off‐mid‐slice rendering in Figure 1b, the in‐plane chord is scaled by the cone‐beam ray‐path factor $L_{3D}/L_{xy}$. FDK Tuy‐condition error is not modelled. The R=13mm cylinder is placed on the rotation axis throughout, except in Figure 5, where it sits at (2.71,−5.53) mm to match a representative mouse specimen.^24^

**FIGURE 1:**
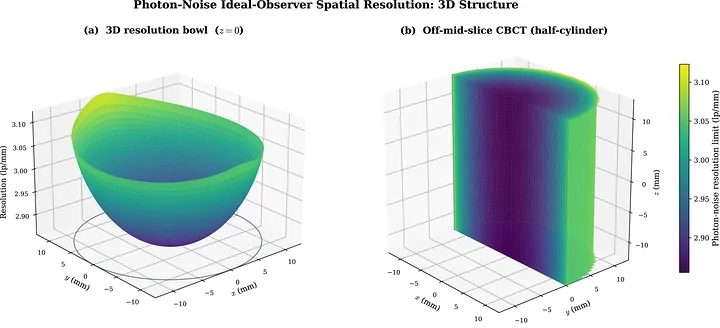
Three‐dimensional structure of the photon‐noise ideal‐observer resolution limit, at ΔC=500HU inside a centred cylindrical specimen model (R=13mm, half‐height Z=13mm). The Crowther angular‐sampling limit is not included here. The combined limit including it is shown later in Figure 5. (a) 3D resolution bowl on the mid‐plane z=0. Surface height encodes $f_{\text{noise}}$ (lp/mm). The specimen rim is outlined at the floor of the plot. (b) Off‐mid‐slice half‐cylinder rendering: cut plane at y=0 (front face), curved cylinder wall on the y≥0 half (behind), and top and bottom caps at z=±Z. For this scanner's $4^{\circ }$ cone half‐angle the surface is effectively z‐invariant, so the mid‐plane bowl applies throughout the reconstruction volume.

**FIGURE 2:**
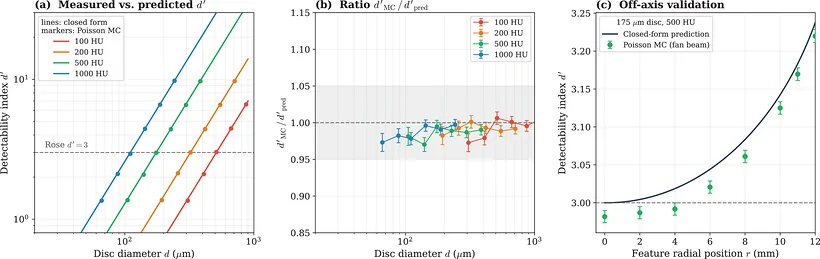
Poisson Monte Carlo validation of the closed‐form ideal‐observer bound on the R=13mm water cylinder at $N_{0}=4203$ photons/pixel, 220 views over $192^{\circ }$. (a, b) On‐axis detectability versus disc diameter at four contrasts, simulation (markers, 10000 realizations per hypothesis. Error bars are the Monte Carlo standard error) against the closed form (lines). The shaded band in (b) is ±5%. (c) Off‐axis validation of Equation 8 for a disc of 175μm diameter at 500HU (100000 realizations per hypothesis): simulated‐to‐predicted $d^{\prime }$ ratio at eight radial positions.

**FIGURE 3:**
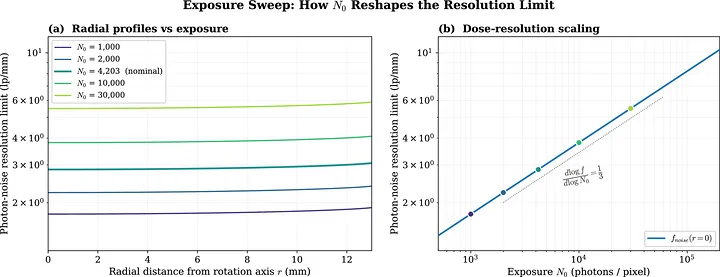
Effect of exposure $N_{0}$ on the photon‐noise resolution limit at ΔC=500HU. (a) Radial profile $f_{\text{noise}}\left(r\right)$ at five log‐spaced exposures. Increasing $N_{0}$ raises every curve by the predicted $N_{0}^{1/3}$ factor without changing its shape. (b) Log‐log dose‐resolution scaling at the rotation axis. The blue curve traces $f_{\text{noise}}\left(r=0\right)$. Colored markers correspond to the five exposures in panel (a). The dotted guideline shows the predicted 1/3 slope.

**FIGURE 4:**
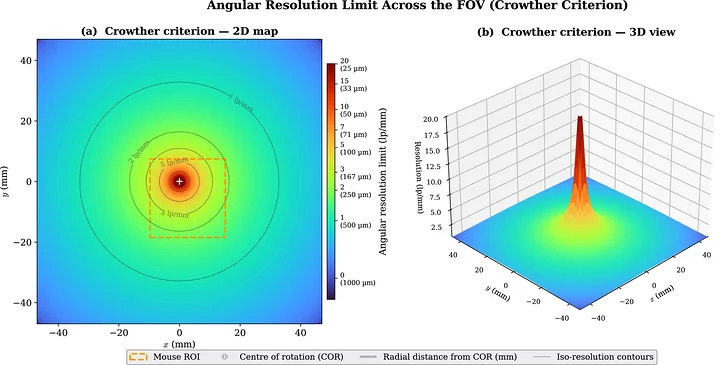
Angular sampling resolution limit (Crowther criterion). (a) 2D heatmap over the FOV, with iso‐resolution contours overlaid in black. (b) Matching 3D surface. The limit is capped at the detector Nyquist (20.1lp/mm). Dashed‐orange box: representative off‐axis mouse ROI. White plus: centre of rotation.

**FIGURE 5:**
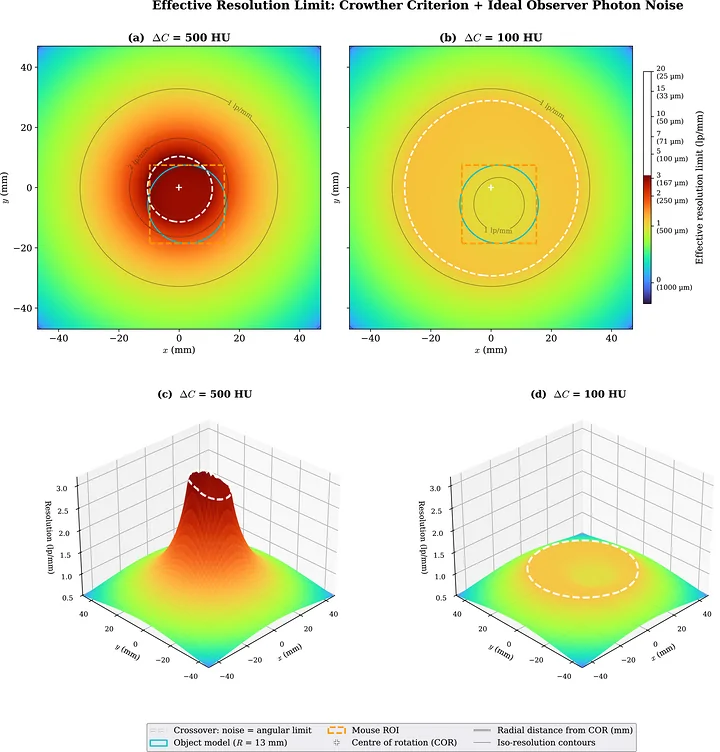
Combined effective resolution limit $f_{\text{eff}}\left(r\right)=\min\left[f_{\text{noise}},f_{\text{Crowther}}\right]$. (a, c) ΔC=500HU. (b, d) ΔC=100HU. Top row: 2D heatmaps with iso‐resolution contours (black) and noise‐Crowther crossover (dashed white). Bottom row: 3D surfaces. Cyan circle: specimen model used in the off‐axis ideal‐observer evaluation. Dashed‐orange box: representative off‐axis mouse ROI.

### 3.4 Parametric sweeps

Two parametric sweeps characterise how the resolution limit responds to scan‐design choices, both on the centred R=13mm specimen model at ΔC=500HU:

- **Exposure sweep**: $N_{0}\in \left\{1000,2000,4203,10000,30000\right\}$ photons/pixel at fixed $N_{\theta }=220$, spanning roughly an order of magnitude either side of the nominal. Crowther is not included in this sweep, because $N_{0}$ does not enter the Crowther formula. The figure (Figure 3) shows only the photon‐noise component.
- **Projection‐count sweep**: $N_{\theta }\in \left\{55,110,220,440\right\}$ at fixed $N_{0}=4203$ photons/pixel, over the same $192^{\circ }$ arc, in factor‐of‐two steps from sparse‐view to well‐sampled. Increasing $N_{\theta }$ widens the Crowther cone (Equation 9) linearly and improves the photon‐noise bound as $N_{\theta }^{1/3}$ in $d_{\min}$ (Equation 4). The figure (Figure 6) shows the combined limit so both effects are visible.

The sweep values are hypothetical inputs to Equations 4 and 9, not acquired scans. Only the nominal point $\left(N_{0},N_{\theta }\right)=\left(4203,220\right)$ corresponds to the physical acquisition protocol. The $192^{\circ }$ short‐scan is used throughout to highlight the asymmetry of the off‐axis maps. A full $360^{\circ }$ acquisition would simply render them symmetric. Each sweep is presented as a two‐panel figure, a 3D rendering of the limit surface(s) over the specimen disc and a radial profile of the limit along the +x axis.

**FIGURE 6:**
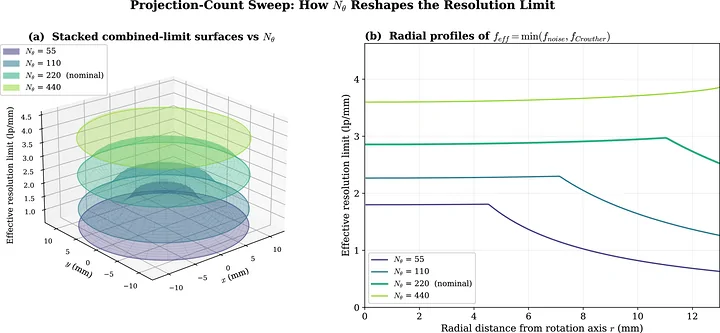
Effect of projection count $N_{\theta }$ on the combined‐limit resolution surface at ΔC=500HU. (a) Stacked 3D surfaces of $f_{\text{eff}}\left(x,y\right)=\min\left(f_{\text{noise}},f_{\text{Crowther}}\right)$ at four projection counts. Surfaces are drawn semi‐transparent so the nesting is visible; lower $N_{\theta }$ (darker) sits inside higher $N_{\theta }$ (lighter). The floor lifts as $N_{\theta }^{1/3}$. The outer rim climbs linearly with $N_{\theta }$. (b) Radial profiles of $f_{\text{eff}}\left(r\right)$ at the same projection counts (linear axes). The noise‐bound central plateau and the Crowther‐bound 1/r tail are visible. The crossover migrates outward with $N_{\theta }$.

### 3.5 Monte Carlo validation of the closed‐form detectability

To validate Equations 4 and 8, we simulated two‐hypothesis (signal‐present / signal‐absent) detection experiments in the projection domain, in a fan‐beam geometry matching the analytical model exactly (R=13mm water cylinder, $N_{0}=4203$ photons/pixel, 220 views over $192^{\circ }$, $\Delta a_{obj}=0.0249\;\mathrm{mm}$, $\mu_{\text{water}}=0.0219\;{\mathrm{mm}}^{-1}$). Expected counts use the exact cylinder chord per ray. Counts are drawn as $N∼\text{Poisson}(N_{0}\;e^{-p})$, log‐transformed, and passed to the prewhitened matched filter of Equation 2, the ideal SKE/BKE observer. The detectability index is measured from the separation of the decision‐variable distributions over 10000 (on‐axis) or 100000 (off‐axis) noise realizations per hypothesis. The on‐axis sweep covers contrasts {100,200,500,1000} HU at six disc diameters bracketing each predicted $d_{\min}$. The off‐axis validation uses a disc of fixed 175μm diameter at 500HU, placed at eight radial positions between the rotation axis and r=12mm, with the local sample pitch of Equation 7.

### 3.6 Anatomical forward projection for the specimen detectability map

To make the specimen detectability map of Section 5.2 quantitative, the per‐pixel transmitted counts are recovered from the real anatomy rather than the uniform‐cylinder model. The FDK mouse slice (75.2μm pixels) is converted to linear attenuation as $\mu =\mu_{\text{water}}\left(1+\mathrm{HU}/1000\right)$, clamped at μ≥0, with $\mu_{\text{water}}=0.0219\;{\mathrm{mm}}^{-1}$ at the effective energy of the 80kV beam. A fan‐beam sinogram of the μ‐map at the scanner geometry of Table 1 (220 views over $192^{\circ }$, rays sampled every 0.03mm) yields the transmitted count $N_{bg}\left(\theta_{j},r\right)=N_{0}\;e^{-p}$ for every image pixel and view, which enters the sum of Equation 8 together with the per‐view local pitch. The identical sum evaluated for the uniform‐cylinder model in the same geometry gives a like‐for‐like comparison.

### 3.7 Presampling MTF measurement

To bound the imaging‐chain blur excluded from the ideal‐data model, we measured the presampling MTF of the scanner using a slanted‐edge analysis, with the blade placed at the isocentre and imaged in a step‐and‐shoot scan at the mouse‐protocol technique of Section 3.1 All values are referred to the object plane. The measured MTF is reported in Section 4.8 and its implications for the closed‐form bounds are discussed in the Limitations (Section 5.3).

## 4 RESULTS

To illustrate the framework, we evaluate it on a representative preclinical micro‐CBCT scanner, the GE eXplore CT 120 of Section 3.1 Except for the flat‐field‐derived $N_{0}$ (Section 3.2) and the numerical validations (Section 4.2), every result below is a closed‐form theoretical evaluation, not an experimental measurement. The specific numerical values in what follows correspond to this acquisition geometry. The closed‐form scaling laws (the $d^{3}$ disc‐detection scaling, the $\Phi^{1/3}$ dose‐resolution law, and the $\sqrt{N_{0}}$ optimal‐allocation ridge) are general results of the framework itself and apply to any scanner.

### 4.1 The photon‐noise resolution limit

We evaluate the closed‐form bound (Equation 4) on a centred uniform water‐equivalent cylinder of radius R=13mm, matching the typical scale of a preclinical mouse specimen on this scanner. At the rotation axis every projection ray traverses the full diameter L=2R=26mm, giving $N_{bg}=N_{0}e^{-\mu_{bg}L}=4203\cdot e^{-0.0219\cdot 26}\approx 2379$ photons/pixel. Table 2 lists the minimum detectable disc diameters. At our nominal acquisition the photon‐noise spatial‐frequency limit at the rotation axis is 0.98lp/mm at ΔC=100HU and 2.86lp/mm at 500HU. Both frequencies sit well below the hardware ceilings of this system in f, the detector Nyquist of 20.1lp/mm and the reconstruction‐grid Nyquist of 6.67lp/mm for our 75μm voxels. Photon noise sets the bound at low contrast, not hardware or the reconstruction grid.

**TABLE 2:** Minimum detectable disc diameter and spatial‐frequency limit at the rotation axis from the projection‐domain ideal observer (Equation 4, $d_{\text{th}}^{\prime }=3$), evaluated on the R=13mm specimen geometry used throughout this work, alongside the Poisson Monte Carlo validation of Section 4.2 (10000 realizations per hypothesis).

ΔC (HU)	Δμ (${\mathrm{mm}}^{-1}$)	$d_{\min}$ (μm)	$f_{\text{noise}}$ (lp/mm)	$d_{\min}$ simulated (μm)	Deviation
100	0.00219	511.9	0.98	514.2	+0.5%
200	0.00438	322.5	1.55	324.3	+0.6%
500	0.01095	175.1	2.86	176.8	+1.0%
1000	0.02190	110.3	4.54	111.3	+0.9%

##### Gap to actual reconstructions

As an illustrative baseline calibration, rather than a definitive benchmark, of how far real algorithms sit from this bound, prior reconstruction studies on the same scanner^1^, ^25^ (the latter being a preprint) report $d_{\min}$ for an open‐source FDK implementation, ASTRA SIRT,^26^ and TIGRE OS‐SART^27^ on a larger image‐quality phantom of different geometry than the specimen model used here (water‐equivalent path L=80mm, R=40mm). Recomputing the ideal‐observer reference at that same geometry, real reconstructions resolve feature diameters roughly 2.8× larger than the bound (equivalently, they reach only ≈1/2.8 of the bound in spatial frequency f), and this factor is approximately independent of contrast, consistent with a structural imaging‐chain penalty rather than a contrast‐specific loss. Part of this gap is that the SKE/BKE matched filter is itself optimistic, requiring exact knowledge of signal location and background that no real observer can satisfy. Gang et al.^19^ quantify the penalty incurred by anatomical clutter in cone‐beam CT, with the size depending strongly on the task and the acquisition geometry. The rest reflects noise‐side propagation in the reconstruction, which closes only with algorithms that better match the matched filter on the noise side, not more dose.

##### Spatial structure of the limit

The closed‐form bound evaluated so far applies only at the rotation axis. The off‐axis fan‐beam extension (Equation 8) generalises it to the full field of view, and the same expression extends naturally to 3D using the cone‐beam chord scaling described in Section 3.3 We apply this on the same R=13mm specimen geometry. Cone‐beam FDK Tuy‐condition error is not modelled, as it is sub‐percent for the $4^{\circ }$ cone half‐angle of this scanner.

Figure 1a presents the photon‐noise resolution as a 3D surface in the central slice z=0. The familiar “bowl” shape is recovered. $f_{\text{noise}}$ is lowest at the specimen centre (r=0), where every projection ray traverses the full diameter and photon attenuation is maximal, and rises toward the rim (r=R), where partial‐chord rays see less attenuation. For this specimen and contrast, $f_{\text{noise}}$ rises from 2.86lp/mm at the centre of the bowl to 3.02lp/mm at the rim. The centre value matches the at‐isocentre closed‐form of Table 2, confirming that the off‐axis formulation reduces to the on‐axis bound.

Figure 1b extends the analysis off the mid‐plane. The half‐cylinder cut renders the resolution on four surfaces of an on‐axis cylindrical specimen of half‐height Z=R=13mm: the cut plane at y=0, the curved cylinder wall on the y≥0 side, and the top and bottom caps at z=±Z. For this scanner the resolution is effectively z‐invariant across the full volume. At (r,z)=(0,±Z) the resolution differs from the central‐slice value at the same r by less than 1%. The mid‐plane analysis therefore applies throughout the reconstruction volume to within sub‐percent error. Scanners with larger cone angles, such as wide‐detector clinical CBCT systems with cone half‐angles of $10^{\circ }$–$15^{\circ }$, would require adding the FDK Tuy‐condition penalty omitted here.

### 4.2 Monte Carlo validation of the closed‐form bounds

Figure 2 compares the closed‐form detectability against the Poisson Monte Carlo simulation of Section 3.5 On‐axis, the simulated $d^{\prime }$ agrees with Equation 3 within 1–3% at all 24 (contrast, diameter) points, and the minimum detectable diameters at the Rose threshold agree within 1% at every contrast (Table 2). Off‐axis, the simulated‐to‐predicted $d^{\prime }$ ratio for Equation 8 is 0.991–0.996 at all eight radial positions, so the spatial shape of the fan‐beam extension is captured essentially exactly. The simulation sits a systematic ∼0.5–1% below the closed form because the Gaussian derivation of Equation 3 drops the second‐order term of the log transform, $E\left[\ln N\right]\approx \ln\bar{N}-1/\left(2\bar{N}\right)$. At $N_{bg}\approx 2379$ behind the cylinder centre this predicts exactly the observed contrast‐independent shortfall. The closed form is thus slightly optimistic, by an amount that shrinks as $1/\bar{N}$, and at the count levels of this scanner the analytic bound is accurate to ∼1% in $d_{\min}$.

### 4.3 Effect of exposure on the photon‐noise limit

Figure 3 characterises how exposure $N_{0}$ reshapes the photon‐noise component of the resolution limit. Figure 3a overlays the radial profile $f_{\text{noise}}\left(r\right)$ for five exposures spanning 1000–30000 photons/pixel at ΔC=500HU. Increasing $N_{0}$ lifts every curve uniformly. Each is a vertical rescaling of the nominal $N_{0}=4203$ profile by the factor ${\left(N_{0}/4203\right)}^{1/3}$. The bowl shape is preserved at every exposure. Only its floor, the minimum of $f_{\text{noise}}\left(r\right)$ at the specimen centre, moves.

Figure 3b plots the central‐slice scaling on log‐log axes. The continuous $f_{\text{noise}}\left(r=0\right)$ curve follows the $N_{0}^{1/3}$ power law of Equation 4. A 30× exposure increase delivers a $\sqrt[{3}]{30}\approx 3.1\times$ resolution gain. The slope guideline confirms the predicted 1/3 log‐log exponent. This is the diminishing‐returns regime of the dose‐resolution trade. Each octave of dose buys only a $\sqrt[{3}]{2}\approx 1.26\times$ resolution improvement.

### 4.4 Spatially varying Crowther criterion

The photon‐noise bound discussed so far is a projection‐domain ceiling that any reconstruction inherits. No algorithm of any kind can resolve a feature whose detectability index falls below threshold on the projection data themselves. Classical, prior‐free reconstructions (FDK, FBP, SIRT, OS‐SART) additionally face the image‐domain Crowther angular‐sampling ceiling (Equation 9), which reconstructions with strong signal priors can evade (Sections 1 and 5.1). The remainder of this section therefore uses the effective limit $f_{\text{eff}}=\min\left[f_{\text{noise}},f_{\text{Crowther}}\right]$ (Equation 10) as the ceiling for classical reconstructions. Figure 4 renders the Crowther criterion alone as a 2D heatmap and matching 3D surface over the FOV. The limit diverges at the rotation axis and falls as 1/r. The dashed‐yellow ROI marks the location of a representative mouse specimen, illustrating that off‐axis specimens see substantially worse angular resolution than ones centred on the isocentre. Across the scanner's circular FOV (radius ≈43.6mm) the Crowther limit ranges from approximately 0.75lp/mm at the rim to the detector‐Nyquist cap of 20.1lp/mm. The interactive web calculator^20^ renders this map for arbitrary $N_{\theta }$, arc coverage, and source distance.

### 4.5 Combined effective resolution limit

Figure 5 combines the off‐axis photon‐noise map with the Crowther limit as $f_{\text{eff}}=\min\left(f_{\text{noise}},f_{\text{Crowther}}\right)$ (Equation 10). The dashed‐white contour marks the noise‐Crowther crossover. At 500HU the noise‐dominated region is a roughly object‐shaped bowl centred on the specimen. Outside it, the Crowther limit takes over and the effective resolution decreases radially. At 100HU photon noise dominates almost the entire FOV. The noise‐limited bowl floor in $f_{\text{eff}}$ has dropped below the Crowther walls everywhere except very near the rotation axis. The 3D surfaces in panels (c) and (d) make the “bowl” shape explicit, a depression in the centre with rising walls outside it.

### 4.6 Parametric sweep: projection count

Unlike exposure, projection count $N_{\theta }$ moves both components of the resolution limit simultaneously. The photon‐noise bound improves as $N_{\theta }^{1/3}$ (Equation 4), and the Crowther bound improves linearly, $f_{\text{Crowther}}\propto N_{\theta }$ (Equation 9). Figure 6 renders both effects in a single picture. Panel (a) stacks the combined‐limit 3D surface $f_{\text{eff}}\left(x,y\right)=\min\left(f_{\text{noise}},f_{\text{Crowther}}\right)$ for four projection counts $N_{\theta }\in \left\{55,110,220,440\right\}$, drawn semi‐transparent so the nesting is visible. Lower $N_{\theta }$ (darker) sits inside higher $N_{\theta }$ (lighter). The floor lifts as $N_{\theta }^{1/3}$ while the Crowther‐dominated outer collar lifts linearly in $N_{\theta }$, so the outer rim climbs faster than the central plateau as projection count grows.

Figure 6b plots the radial profile of $f_{\text{eff}}$ for each $N_{\theta }$. The profiles share the same shape, a noise‐bound central plateau and a Crowther‐bound 1/r tail at large r. The location and height of every feature scales with $N_{\theta }$, as summarised in Table 3. At the lowest $N_{\theta }=55$, the crossover from noise‐ to Crowther‐bound sits at r≈4.5mm, well inside the 13mm specimen, so most of the FOV is angular‐limited. As $N_{\theta }$ grows, the crossover migrates outward, reaching r≈7.1mm at $N_{\theta }=110$, ≈11.0mm at $N_{\theta }=220$ (nominal, still inside the specimen), and at $N_{\theta }=440$ the crossover has passed the rim. The whole specimen interior is now photon‐noise‐bound. Below this transition, $f_{\text{eff}}\left(r=R\right)$ scales linearly with $N_{\theta }$ (pure Crowther). Above it, scaling reverts to the $N_{\theta }^{1/3}$ photon‐noise law.

**TABLE 3:** Combined‐limit summary across the projection‐count sweep at ΔC=500HU, R=13mm. “Crossover r” is the radius at which $f_{\text{noise}}=f_{\text{Crowther}}$. The dash for $N_{\theta }=440$ indicates the crossover lies outside the specimen, so the entire interior is noise‐bound.

$N_{\theta }$	$f_{\text{noise}}\left(r=0\right)$ (lp/mm)	Crossover r (mm)	$f_{\text{eff}}\left(r=R\right)$ (lp/mm)	Binding at rim
55	1.80	4.5	0.63	Crowther
110	2.27	7.1	1.26	Crowther
220	2.86	11.0	2.53	Crowther (nominal)
440	3.60	—	3.86	photon noise

The two‐effect scaling makes $N_{\theta }$ a more efficient parameter than $N_{0}$ for raising low‐contrast resolution, especially while the FOV is still Crowther‐bound. Doubling $N_{\theta }$ improves the central resolution by $\sqrt[{3}]{2}\approx 1.26\times$ and doubles the resolution at every Crowther‐bound radius. Doubling $N_{0}$ only delivers the first of these gains. Once the entire FOV crosses into the noise‐bound regime (which happens for our 13mm specimen at $N_{\theta }\approx 288$ at this exposure), the two parameters become equivalent at every radius.

### 4.7 Optimal photon allocation under a fixed dose budget

The exposure (Section 4.3) and projection‐count (Section 4.6) sweeps each vary one parameter while holding the other fixed. The scan designer is more often interested in the dual question. Given a fixed total photon budget per detector pixel

(12) $$\Phi \;\equiv \;N_{0}\cdot N_{\theta },$$

how should that budget be split between $N_{0}$ and $N_{\theta }$? The optimum follows analytically.

The figure of merit is the worst‐case effective resolution inside a target field of view of radius $r^{*}$,

(13) $$\begin{array}{ccc} f_{\min}\left(r^{*}\right)\; & = & \;\min_{r\;\leq \;r^{*}}f_{\text{eff}}\left(r\right)\;=\;\min_{r\;\leq \;r^{*}}\min[\;f_{\text{noise}}\left(r\right),\\ & & f_{\text{Crowther}}\left(r\right)\;]. \end{array}$$

Equation 13 collapses to a two‐term expression because $f_{\text{noise}}\left(r\right)$ is smallest at r=0 (every projection ray traverses the full diameter there, and photon attenuation is maximal), and $f_{\text{Crowther}}\left(r\right)$ is smallest at $r=r^{*}$ (the Crowther limit falls as 1/r). Therefore

(14) $$f_{\min}\left(r^{*}\right)\;=\;\min\left[\;f_{\text{noise}}\left(0\right),\;f_{\text{Crowther}}\left(r^{*}\right)\;\right].$$

#### The dose‐resolution law

At the rotation axis, every projection sees the same chord L=2R, so the geometric factor in Equation 8 collapses to a constant and Equation 4 reduces to

(15) $$d_{\min}\left(0\right)\;=\;{\left(\frac{3\;d_{\text{th}}^{\prime 2}\;\Delta a_{obj}}{2\;\Phi \;e^{-2\mu R}\;\Delta \mu^{2}}\right)}^{\;1/3}.$$

The product $N_{0}N_{\theta }$ enters as the single combination Φ. Inverting to the spatial‐frequency bound and defining the constant

(16) $$K\;\equiv \;\frac{1}{2}{\left(\frac{2\;e^{-2\mu R}\;\Delta \mu^{2}}{3\;d_{\text{th}}^{\prime 2}\;\Delta a_{obj}}\right)}^{\;1/3},$$

gives

(17) $$f_{\text{noise}}\left(0\right)\;=\;K\cdot \Phi^{1/3}.$$

The photon‐noise resolution floor $f_{\text{noise}}\left(0\right)$ at the centre of the specimen depends only on the total photon budget, not on how it is split between $N_{0}$ and $N_{\theta }$. For our scanner at ΔC=500HU inside an R=13mm specimen, K=0.02932 lp/mm· ${\mathrm{photons}}^{-1/3}$.

#### The optimum

With $f_{\text{noise}}\left(0\right)$ fixed by Φ, the only handle on $f_{\min}\left(r^{*}\right)$ is $f_{\text{Crowther}}\left(r^{*}\right)=N_{\theta }/\left(2\Delta \theta \;r^{*}\right)$, which grows linearly with $N_{\theta }$. Below threshold, Crowther binds and $f_{\min}$ grows linearly in $N_{\theta }$. Above threshold, photon noise binds and $f_{\min}$ plateaus. The threshold is set by equating the two:

(18) $$N_{\theta }^{★}\left(r^{*},\Phi \right)\;=\;2\;K\;\Delta \theta \;r^{*}\cdot \Phi^{1/3}.$$

The maximum achievable worst‐case resolution is then

(19) $$\begin{array}{c} \;f_{\min}^{\max}\left(r^{*},\Phi \right)\;=\;K\cdot \Phi^{1/3},\; \end{array}$$

attained for any $N_{\theta }\geq N_{\theta }^{★}$. The corresponding per‐pixel exposure is $N_{0}^{★}=\Phi /N_{\theta }^{★}=\Phi^{2/3}/\left(2K\Delta \theta r^{*}\right)$.

#### Visualisation

Figure 7 renders the full design space at $r^{*}=R$. Figure 7a is the 3D surface $f_{\min}\left(N_{0},N_{\theta }\right)$ with the two axes log‐spaced. Three iso‐Φ trajectories (constant total dose, in red/orange/purple for $\Phi \in \{10^{5},10^{6},10^{7}\}$) are drawn directly on the surface: each climbs the Crowther ramp at low $N_{\theta }$ and plateaus once it crosses the ridge into the noise‐bound region. The plateau heights are spaced by exactly $10^{1/3}\approx 2.15$ in resolution, the dose‐resolution law made visible. Figure 7b is the top‐down 2D heatmap of the same data with the design overlays added:

- **Iso‐** Φ **lines** (dashed, slope −1 in log‐log): trajectories of constant total dose. The scan designer's degree of freedom is to slide along one of these lines.
- **Optimal‐allocation ridge** (gold, slope +1/2 in log‐log): $N_{\theta }^{★}\propto \sqrt{N_{0}}$ for fixed $r^{*}$, derived from Equation 18 by eliminating Φ. Below the ridge, Crowther binds. Above the ridge, photon noise is saturated. Walking along any iso‐Φ line until it meets the ridge gives the optimal split for that dose.
- **Iso‐** $f_{\min}$ **contours** (white line): constant‐resolution surfaces. They are vertical (Crowther: $f_{\min}$ depends only on $N_{\theta }$) below the ridge and curve toward the diagonal (noise: $f_{\min}$ depends on $N_{0}N_{\theta }$) above it.
- **Nominal acquisition** (red star): $\left(N_{0},N_{\theta }\right)=\left(4203,220\right)$, sitting visibly below the ridge, confirming that our actual scan is Crowther‐limited at the rim of the specimen.

**FIGURE 7:**
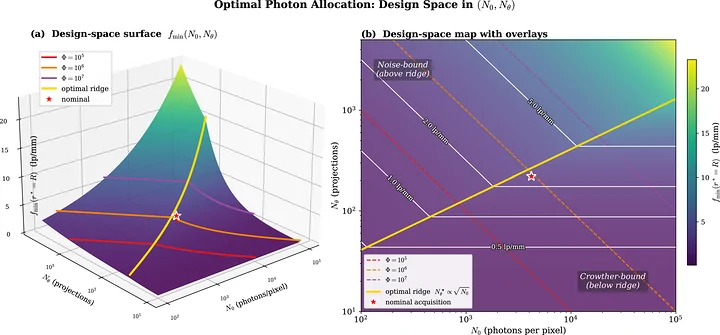
Dose‐allocation design space at ΔC=500HU, $r^{*}=R=13\;\mathrm{mm}$. (a) 3D surface $f_{\min}\left(N_{0},N_{\theta }\right)$ with both axes log‐spaced. Three iso‐Φ trajectories (red, orange, purple for $\Phi =10^{5},10^{6},10^{7}$) climb the Crowther ramp and plateau in the noise‐bound regime. The red star is our nominal acquisition. (b) 2D heatmap of the same surface with design overlays: iso‐Φ lines (dashed, slope −1), the optimal‐allocation ridge $N_{\theta }^{★}\propto \sqrt{N_{0}}$ (gold solid, slope +1/2), iso‐$f_{\min}$ contours (white line), and the two regimes labelled. The nominal acquisition sits just below the ridge in the Crowther‐bound half.

#### Practical rules

Three rules follow directly from Equations (17), (18), (19):

1. **The dose‐resolution law is cubic for the disc task**. $f_{\min}^{\max}\propto \Phi^{1/3}$, so each doubling of worst‐case resolution requires 8× the total photons. This is the classical resolution‐dose trade in CT,^28^, ^29^ here recovered as a property of the ideal observer rather than of any specific reconstruction. The exponent follows from the $d^{3}$ scaling of the disc detectability integral in Equation 3, and other feature geometries yield different exponents.
2. $N_{\theta }^{★}$ **scales linearly with FOV radius**. Larger ROIs demand more views in linear proportion. Imaging a 4× larger radius at the same maximum resolution requires 4× the views.
3. **Add views when** $N_{\theta }<N_{\theta }^{★}$. **Add flux per view when** $N_{\theta }>N_{\theta }^{★}$. Adding $N_{0}$ when $N_{\theta }<N_{\theta }^{★}$ does not improve the worst‐case resolution because Crowther is binding. Symmetrically, adding $N_{\theta }$ when $N_{\theta }>N_{\theta }^{★}$ does not help because photon noise is saturated. The optimal scan operates exactly at $N_{\theta }^{★}$.

#### Application to our acquisition

As a worked example, take our nominal acquisition with Φ=924660 and $r^{*}=R=13\;\mathrm{mm}$. The optimum is $N_{\theta }^{★}\approx 250$ views and $N_{0}^{★}\approx 3700$ photons/pixel. Our actual acquisition uses $N_{\theta }=220$ and $N_{0}=4203$, putting us slightly on the Crowther‐bound side of the line but very close to it. Redistributing the same total dose toward $N_{\theta }^{★}$ would improve the rim resolution from $f_{\text{Crowther}}\left(R\right)=2.52\;\mathrm{lp}/\mathrm{mm}$ to $f_{\min}^{\max}=2.86\;\mathrm{lp}/\mathrm{mm}$, a ∼14% gain rather than the factor‐of‐two‐plus gap that would exist at larger specimen radii. The constraint we omit is the detector noise floor. At very low $N_{0}$ per pixel, electronic noise stops the Poisson assumption from holding, and Equation 15 ceases to apply.

### 4.8 Measured presampling MTF

At the object plane, the slanted‐edge measurement of Section 3.7 gives ${\mathrm{MTF}}_{50}=1.55\;\mathrm{lp}/\mathrm{mm}$ and ${\mathrm{MTF}}_{10}=7.5\;\mathrm{lp}/\mathrm{mm}$, with a modulation of 0.66–0.22 across the band f=1.0–4.5lp/mm spanned by the reported $d_{\min}$ of 512–110μm. At the operating exposure the detector therefore transfers the entire photon‐supported band, so photon statistics rather than detector blur is the practically binding constraint. Only above $N_{0}\approx 1.9\times 10^{4}$ photons/pixel would the measured MTF, not photon noise, become binding.

### 4.9 Interactive web calculator

The closed‐form bounds derived above are implemented in an open‐source browser‐based calculator^20^ released with this work. It accepts arbitrary scanner geometries (any $N_{0}$, $N_{\theta }$, arc coverage, source‐to‐isocentre distance, contrast, and specimen geometry) and renders the photon‐noise (Equations (4), (5), (6), (7), (8)), Crowther, and combined‐limit maps. The calculator is available at https://ubc‐ford‐lab.github.io/ideal_observer_SKE_BKE_model/ and its source code at https://github.com/UBC‐Ford‐lab/ideal_observer_SKE_BKE_model.

## 5 DISCUSSION

### 5.1 Scope of each limit and learned‐prior reconstructions

The two components of $f_{\text{eff}}$ are not the same kind of object. $f_{\text{Crowther}}$ is a Fourier‐domain sampling cap. Above this frequency, no Fourier component can be recovered from $N_{\theta }$ angular samples without aliasing. It is signal‐independent and purely geometric. $f_{\text{noise}}=1/\left(2\;d_{\min}\left(\Delta \mu \right)\right)$ is a detection threshold for a specific feature (a disc of contrast Δμ, at the Rose $d^{\prime }=3$ threshold), signal‐dependent and SNR‐conditioned rather than a frequency cutoff in the strict Fourier sense. The two share units of lp/mm but mean different things. Converting smallest detectable feature size to a frequency via $f=1/(2\;d_{\min})$ is itself a convention. Two independent necessary conditions bind, $f\leq f_{\text{noise}}$ from photon statistics (Section 2.1) and $f\leq f_{\text{Crowther}}$ from angular sampling (Section 2.2). Their pointwise minimum is the more restrictive of the two at each location. The combined map is a heuristic summary, not a unified theoretical bound. Each component remains rigorous within its own scope.

Photon noise is thus universal (a property of the projection data themselves), while Crowther is specific to reconstructions without strong signal priors. First, the gap between actual reconstructions and the ideal‐observer bound (Section 4.1) is the room available for improvement on a fixed dataset. Closing it requires algorithms that match the matched filter on the noise side, not more dose. Second, the Crowther line in Figure 5 should not be read as a strict ceiling for every reconstruction class. Learned reconstructions that incorporate signal priors^11^, ^12^, ^13^, ^14^, ^15^ have been demonstrated to recover frequencies above $f_{\text{Crowther}}$ in sparse‐view CT. The cost is the assumption that the prior is correct for the signal at hand. Where it fits, the assumption is benign. For out‐of‐distribution anatomy or novel pathology, there is no such guarantee. The photon‐noise component alone is the binding floor that applies regardless of prior strength. The matched filter is already optimal for the fixed SKE/BKE hypothesis, so a learned method that appears to surpass it is importing outside information or answering a different, easier task. More broadly, because the binding limit varies spatially across the field of view, the absolute resolution numbers reported here should be read as approximations. The framework is most useful for relative comparison between reconstructions on a fixed dataset, where the algorithm is the only variable.

### 5.2 Scan‐design implications

The parametric sweeps in Sections 4.3, 4.4, 4.5, 4.6 translate into the following design rules. For an experimenter constrained by total dose $D_{\text{tot}}\propto N_{0}\cdot N_{\theta }$:

- If photon noise dominates over the region of interest (low contrast, central placement of the specimen), both $N_{0}$ and $N_{\theta }$ buy resolution as $\propto D_{\text{tot}}^{1/3}$. The choice between more views and brighter views is then governed by mechanical / electronic constraints (gantry stability, detector saturation, electronic noise floor), not by the resolution bound itself.
- If angular sampling dominates (high contrast, peripheral specimen placement), $N_{\theta }$ is strictly preferred to $N_{0}$. It improves both bounds, while $N_{0}$ improves only the noise bound that is not binding.

For our scanner with low‐contrast targets, the binding limit is photon noise everywhere in the typical specimen volume (Figure 5b), so the practical takeaway is that low‐contrast resolution is dose‐limited, not geometry‐limited, on this system at the nominal acquisition. Centring the specimen on the rotation axis is still recommended, because it keeps the angular limit safely above the noise limit, but the gains are second‐order at low contrast.

Because $f_{\text{eff}}\left(x,y\right)$ is defined everywhere in the field of view, the bound can be sampled at the actual pixel locations of a reconstructed specimen to give a resolution map over real anatomy. Figure 8 applies this to an FDK reconstruction of a representative mouse, with the combined limit overlaid transparently in two contrast variants and the anatomical‐versus‐cylinder comparison in a third panel.

**FIGURE 8:**
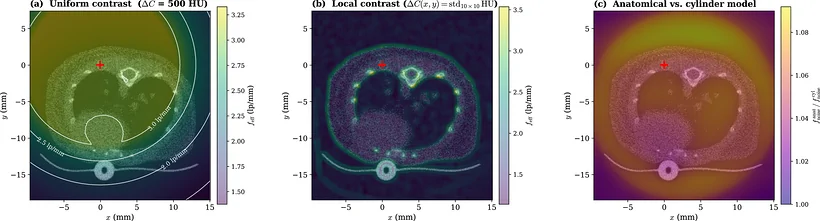
The combined limit $f_{\text{eff}}$ evaluated at every pixel of an FDK mouse reconstruction (75.2μm pixels), with photon counts taken from the forward‐projected anatomical μ‐map (Section 3.6). (a) Fixed contrast ΔC=500HU everywhere, with iso‐resolution contours. $f_{\text{eff}}$ spans 1.38–3.33 lp/mm. (b) Local contrast, ΔC(x,y) being the HU standard deviation over a 10×10 pixel window (floor 5HU). Bone, lung–air interfaces, and tubing reach high $f_{\text{eff}}$. (c) Anatomical over uniform‐cylinder photon‐noise limit, 1.03–1.08 over tissue, so the cylinder model is conservative everywhere. Red cross marks the rotation axis. $N_{\theta }=220$ over $192^{\circ }$.

#### Quantitative anatomical grounding

The map is quantitative, not merely illustrative. The photon‐noise component in Figure 8 uses the true per‐pixel, per‐view transmitted counts $N_{bg}\left(\theta_{j},r\right)$ from the forward‐projected anatomical μ‐map (Section 3.6) rather than the uniform‐cylinder model. Panel (c) quantifies how much that idealisation mattered. The real anatomy shifts the photon‐noise limit by only +3 to +8% over tissue (median +5.5%), always in the favourable direction, because the circumscribing cylinder over‐states attenuation along essentially every ray. The uniform‐cylinder evaluations used throughout this paper are therefore uniformly conservative for this specimen class. This robustness comes from the framework itself. The 220‐view sum averages per‐view anatomical variation, and the cube‐root scaling from photons to resolution means a 17% photon‐count difference moves the limit by only ∼5%. With that scope in mind, Figure 8a evaluates $f_{\text{eff}}$ at a fixed contrast ΔC=500HU everywhere, isolating the geometric and noise structure of the bound itself. With the anatomical $N_{bg}$, $f_{\text{noise}}$ at the isocentre is 3.05lp/mm and the map spans 1.38–3.33 lp/mm across the FOV, the variation set by path attenuation and radial distance from the rotation axis.

Figure 8b goes further. At each pixel, the contrast input to the formula is set to the local intensity standard deviation $\Delta C\left(x,y\right)={\mathrm{std}}_{w}\left[\mathrm{HU}\right]\left(x,y\right)$ computed in a small w=10×10 pixel (≈0.75mm) window. This local standard deviation is a practical local‐contrast proxy, not the exact SKE/BKE Δμ of a specified feature. The interpretation is anatomically grounded. ΔC(x,y) is the contrast scale of features actually present in the local neighbourhood, so the map answers “can the features that live at this pixel be resolved here?” rather than the under‐specified “what is the resolution at this pixel?”. Resolution is contrast‐dependent in CT. No single number exists without a contrast specification. The map highlights edges and high‐frequency anatomical structures. Bone surfaces, lung‐air interfaces, and the small tubing at the bottom of the FOV glow at high $f_{\text{eff}}$, while homogeneous soft‐tissue interiors sit lower because the local contrast scale there (dominated by reconstruction noise, ∼75HU) is much smaller than at edges. A pixel‐by‐pixel map answers questions that a single MTF number cannot, such as which anatomical region to use for quantitative endpoints, where to place an ROI for a measurement that needs a specific feature size, and whether a lesion of known contrast and location is detectable in the data at hand.

### 5.3 Limitations and unmodelled sources of resolution loss

The closed‐form bound keeps only two limits and ignores several physical effects that contribute to real‐world resolution loss. For each, we state the direction in which it would shift the bound.

#### SKE/BKE optimism

The ideal observer assumes the signal location and background are known exactly. Real detection tasks face structured anatomy, “anatomical noise”, that our model does not include. The generalised detectability analysis of Gang and colleagues,^19^ building on Hanson's foundational detectability work,^2^ shows that anatomical clutter can degrade detectability substantially in cone‐beam CT, with the magnitude depending on the task, the clutter spectrum, and the acquisition geometry. The bound here is an upper limit on detectability. Real performance, even for an ideal observer, will be worse than reported.

#### Electronic noise floor and polyenergetic spectrum

The Poisson assumption breaks at very low per‐pixel photon counts where electronic noise dominates. Section 4.7 mentions this as the floor on the $N_{0}$ side of any dose redistribution. The polyenergetic X‐ray spectrum and beam hardening additionally make μ depend on path length. For our setup this affects $N_{bg}$ by a few percent and does not change the scaling laws.

#### Cone‐beam Tuy‐condition error

The 2D fan‐beam off‐axis formula (Equation 8) neglects the FDK approximation error that grows with cone angle. For our $4^{\circ }$ cone half‐angle Section 4.1 shows this is sub‐percent. Wide‐detector clinical CBCT geometries with $10^{\circ }$–$15^{\circ }$ cone would require an explicit correction.

#### Unmodelled imaging‐chain effects

The measured presampling MTF (Sections 3.7 and 4.8) shows that none of the effects excluded from the model tightens the bound. Gantry‐motion and finite‐pulse‐width smearing vanish under the step‐and‐shoot acquisition (the gantry is stationary during each exposure). Focal‐spot penumbra (≈37μm at the object plane for the 0.3mm spot) and detector blur are jointly captured by the measured MTF. Detector lag is negligible for this optically coupled CCD (<2μm tangential smear). Scattered photons add counting noise but no task signal, so at worst they divide the effective photon count by (1+SPR),^30^ which the cube‐root scaling of Equation 4 compresses to ${\left(1+\mathrm{SPR}\right)}^{1/3}$ on $d_{\min}$. At the ≈0.2 ratio measured for a mouse‐sized phantom in cone‐beam micro‐CT at 45kV,^31^ an over‐estimate for the harder 80kV beam used here, this is at most $1.2^{1/3}\approx 6\%$. Each of these degrades a real system relative to the ideal‐data model, so the closed‐form values remain valid lower bounds on $d_{\min}$.

#### Relation to the cascaded‐systems / NEQ framework

A more rigorous treatment of all the above effects together is the Fourier‐domain cascaded‐systems analysis of Rabbani, Shaw & Van Metter,^16^ Cunningham, Westmore & Fenster,^17^ and Tward & Siewerdsen,^18^ which derives a generalised noise‐equivalent quanta NEQ(f) accounting for photon statistics, detector blur (MTF T(f)), focal‐spot blur, additive electronic noise (NPS $S_{e}\left(f\right)$), and reconstruction filtering. The closed‐form $d_{\min}$ used here is the projection‐domain matched‐filter limit of that machinery, evaluated before detector blur, electronic noise, and reconstruction filtering enter the chain. In this limit, NEQ(f) reduces to the input photon count $N_{0}$, and the matched‐filter detectability recovers our $d^{3}$ disc‐detection scaling, consistent with the cascaded results of Gang et al.^19^ Detector MTF rolloff at the spatial frequencies the task depends on, electronic‐noise dominance at very low $N_{0}$ per pixel, and reconstruction‐filter shaping are captured by full cascaded‐NEQ evaluation and should be used when these effects are non‐negligible. The closed forms suit scan‐design trade‐off questions, and a further advantage over a full cascaded‐NEQ evaluation is that their spatially varying structure can be evaluated and displayed directly, as in the combined‐limit maps above. Absolute predictions in regimes where any of these effects matter should be cross‐checked against the cascaded NEQ value.

## 6 CONCLUSION

Spatial resolution in preclinical cone‐beam micro‐CT is bounded by two reconstruction‐independent limits. A photon‐noise limit applies to any reconstruction algorithm. An angular‐sampling limit applies to reconstructions without signal priors. The former is a task‐based detectability threshold expressed in frequency units, not a Fourier cutoff, and the two combine, as a practical summary of two independent necessary constraints, into a spatially varying resolution surface that depends only on scan parameters and feature contrast, not on the choice of reconstruction. For our GE eXplore CT 120 on a typical mouse‐specimen geometry (R=13mm), the photon‐noise limit at the rotation axis is 0.98lp/mm at ΔC=100HU and 2.86lp/mm at 500HU. Classical FDK and iterative reconstructions resolve only features several times larger than this bound allows, leaving headroom for noise‐side algorithmic improvement. Reconstructions that incorporate signal priors may exceed the Crowther limit at the cost of relying on those priors. They cannot exceed the photon‐noise limit at any cost.

## CONFLICT OF INTEREST STATEMENT

The authors declare no conflicts of interest.

## ETHICS STATEMENT

No animal experiments were conducted as part of this study. The in vivo mouse data used here were collected in a previously published thoracic FLASH radiotherapy study^23^, ^24^ under a protocol approved by the University of British Columbia Animal Care Committee (Protocol No. A21‐0060, approved August 31, 2021), performed in accordance with the guidelines of the Canadian Council on Animal Care and the ARRIVE guidelines.^32^ The imaging data were re‐used here for reconstruction‐bound evaluation only. No animals were imaged, handled, or subjected to any procedures as part of this work.

## USE OF GENERATIVE‐AI TOOLS

Claude (Anthropic) was used in some places to assist with manuscript preparation. All AI‐generated content was reviewed, verified, and revised by the authors, who take full responsibility for the final manuscript.

## Data Availability

The interactive web calculator and its source code are publicly available at https://ubc‐ford‐lab.github.io/ideal_observer_SKE_BKE_model/ and https://github.com/UBC‐Ford‐lab/ideal_observer_SKE_BKE_model, respectively.^20^ The figure‐generation scripts and scan data are available from the corresponding author on reasonable request.
